# Economic importance, taxonomic representation and scientific priority as drivers of genome sequencing projects

**DOI:** 10.1186/s12864-016-3100-9

**Published:** 2016-11-11

**Authors:** Geneviève C. Vallée, Daniella Santos Muñoz, David Sankoff

**Affiliations:** Department of Mathematics and Statistics, University of Ottawa, 585 King Edward Avenue, Ottawa, K1N 6N5 Canada

**Keywords:** Genome sequencing, Crop plants, Model organisms

## Abstract

**Background:**

Of the approximately two hundred sequenced plant genomes, how many and which ones were sequenced motivated by strictly or largely scientific considerations, and how many by chiefly economic, in a wide sense, incentives? And how large a role does publication opportunity play?

**Results:**

In an integration of multiple disparate databases and other sources of information, we collect and analyze data on the size (number of species) in the plant orders and families containing sequenced genomes, on the trade value of these species, and of all the same-family or same-order species, and on the publication priority within the family and order. These data are subjected to multiple regression and other statistical analyses. We find that despite the initial importance of model organisms, it is clearly economic considerations that outweigh others in the choice of genome to be sequenced.

**Conclusions:**

This has important implications for generalizations about plant genomes, since human choices of plants to harvest (and cultivate) will have incurred many biases with respect to phenotypic characteristics and hence of genomic properties, and recent genomic evolution will also have been affected by human agricultural practices.

## Background

Genome sequencing has provided researchers with valuable insight into the evolution and genetic structure of many organisms. In 2000, the first plant genome *Arabidopsis thaliana* was sequenced [1]. This plant, of no agricultural or other economic interest, was chosen by the scientific community purely on the basis of its long-term status as a model organism for botanists and technical considerations related to the projected ease of sequencing. Two years later the *Oryza sativa* (rice) genome was published [2, 3]. In contrast to *Arabidopsis*, rice has enormous agricultural significance. Today around two hundred plant genome sequences have been published and many more are in the process of being sequenced and published. Though the cost of the sequencing itself has dropped considerably, genome sequence projects remain costly because of the preliminary steps, quality control, gene annotation and data-basing, all of which require considerable investment. At the same time, it is increasingly difficult to publish a genome sequence in the top science journals. It is of interest, for both pure and applied science, to understand what drives the choice of species to be sequenced. To what extent is it a question of surveying the nature and evolution of genomes across the plant tree of life? Or to sequence a genome in a previously unexplored family or order? And to what extent is it to furnish aid to breeders, growers, geneticists, plant pathologists and industry in improving the quality, quantity and other properties of economically important species. This paper attempts to answer these questions by documenting the taxonomic distribution of sequenced genomes as a function of species abundance within a taxon, the taxonomic novelty of species’ genome and the total economic value of species within a taxon.

There is a longstanding tension between the efforts to prioritize purely scientific interests versus applied and commercial demands in the choice of species to which we devote scarce research resources. This predates genomic science by many decades, but is well encapsulated in this 2002 plea to continue the focus on *Arabidopsis*:

“Why Arabidopsis? Why not concentrate our research efforts and resources on a species that will actually provide food for our world or useful products for industrial uses? In order to make the strides necessary to increase crop production in a relatively short time, we have to be able to move forward quickly and spend the available human and financial resources as efficiently as possible. This is the advantage of a model system: an organism that is easily manipulated, genetically tractable, and about which much is already known. By studying the biology of Arabidopsis, the model plant, we can gain comprehensive knowledge of a complete plant. In the laboratory, Arabidopsis offers the ability to test hypotheses quickly and efficiently. With the knowledge we gain from the model plant thus established as a reference system, we can move forward with research and rapidly initiate improvements in plants of economic and cultural importance” [4].

Similar arguments were made for *Mimulus*, *Medicago*, *Brachypodium* and other model plant genome sequence projects initiated relatively early on.

At the same time there is no impugning the urgency of sequencing crop plants for breeders, growers, plant pathologists and industry in order to improve the quality, quantity and other properties of economically important species, and to prevent and alleviate famine and malnutrition in developing countries [5]. A few researchers have acknowledged that most plant genomes that have been sequenced to date are crop genomes [6, 7]. However, the relationship between the plant genomes that are chosen to be sequenced and their pure scientific interest versus agricultural, horticultural, forestry or medicinal relevance has not previously been studied quantitatively.

## Methods

Three types of data were required for this research: species abundance within angiosperm (and conifer) taxa at various levels, total annual value worldwide of plant products, by species, and a list of species whose genome sequence has been published. Our initial data on plants that have been sequenced was collected from the National Centre for Biotechnology Information (NCBI). This list was not comprehensive, since plants whose genomes had been sequenced recently at the time of the data collection (spring of 2015), such as *Ananas comosus* (pineapple) [8], *Coffea canephora* (Robusta coffee) [9], *Musa balbisiana* (wild banana) [10], and *Utricularia gibba* (humped bladderwort) [11] were not present in the NCBI list. We added as many of these we could find to our list and included them in the analysis. We have continued updating to May 2016.

In all, we found 202 distinct species whose genome had been sequenced; however, only 172 were useful to the present study. All algae and mosses were dropped, due to the lack of any economic data. The remaining species, confined to the flowering plants (angiosperms) and the conifer order of gymnosperms, were classified by taxonomic class or subclass, order, family and genus, based on the APG III system of flowering plant classification [12]. The APG system was chosen rather than the Cronquist or other system [13], since it is continually updated to reflect recent plant DNA evidence and other data.

This dataset is available at: http://216.48.92.133/Softwares/PlantGenomes/index.htm. As more plant genomes are sequenced, more families and orders will be included. A fragment of this dataset is depicted in Table 1.

Next, economic data relating to agricultural and forestry products was collected. For agricultural products, this data was compiled from the Food and Agriculture Organization of the United Nations [14]. For agricultural production, the most recent data on economic value is dated from 2013. This data is presented in current US dollars.

Data on forestry products was compiled from a United Nations Economic Commission for Europe Timber Division report on the forestry industry published in 2006 [15]. The data included information on roundwood and sawnwood, for both conifers and non-conifer trees. The conifer section included data on pine, fir, and spruce, and information on birch, beech, poplar, and oak was found in the non-conifer section. Unfortunately, the data dated back to 2004 and only included select countries, notably European and North American. More recent world data for total roundwood and sawnwood production did not provide a breakdown by tree type. The UNECE/FAO Timber Division report provided exports for each country and from this data we aggregated across all countries the total value by each type of tree. This was done for both the sawnwood and roundwood data, and then summed for a grand total for each tree type. This number was then used as the economic value for each type of tree.

After having collected the economic value for all agricultural (including horticultural and other uses) and forestry products, we classified all sequenced species taxonomically according to APG III. For analytical purposes, we retained only order and family, as class/subclass was not of high enough resolution for meaningful analysis, while genus was too high a resolution, since for almost all the species we studied no economic data distinguished between species in the same genus. Once all products were classified, we calculated an aggregate value of for each family and order. Note that some species of economic value belong to a family and even to an order containing no genome-sequenced species when these data were collected.

The data on the total number of species in all of the families and orders was collected from The Plant List [16] and the Encyclopaedia Britannica [17], respectively.

**Table 1:** Fragment of data on species, family, order and year sequenced

Species	Common Name	Family	Order	Year
⋮				
Azadirachta indica	Neem	Meliaceae	Sapindales	2012
Beta vulgaris	Sugar Beet	Amaranthaceae	Caryophyllales	2014
Betula nana	Alpine Birch	Betulaceae	Fagales	2013
Brachypodium distachyon	Brachypodium	Poaceae	Poales	2010
Brassica napus	Rape	Brassicaceae	Brassicales	2003
Brassica oleracea	Cabbage/Cauliflower	Brassicaceae	Brassicales	2011
Brassica rapa	Field Mustard	Brassicaceae	Brassicales	2011
Cajanus cajan	Pigeon Pea	Fabaceae	Fabales	2011
Camelina sativa	False Flax	Brassicaceae	Brassicales	2013
Cannabis sativa	Hemp	Cannabaceae	Rosales	2011
Capsella rubella	Caspella	Brassicaceae	Brassicales	2013
Capsicum annuum	Cayenne Pepper	Solanaceae	Solanales	2014
Carica papaya	Papaya	Caricaceae	Brassicales	2008
Carthamus tinctorius	Safflower	Asteraceae	Asterales	2016
Castanea mollissima	Chinese Chestnut	Fagaceae	Fagales	2011
Catharanthus roseus	Madagascar Periwinkle	Apocynaceae	Gentianales	2013
⋮				

From these data, we constructed Table 2, reflecting all the families found from both the agricultural and forestry products data, as well as from the list of plants sequenced. Only families containing species that have been sequenced, or have economic value, are included. Similarly, Table 3 was constructed for taxonomic orders. Almost a half of all angiosperm and gymnosperm plant orders, but less than a sixth of all families are present in these tables.

**Table 2:** Data set on families, including species abundance, economic value, and number of sequenced genomes

		Total value					Total	
Family	Species	(Million $)	Seqs.		Family	Species	Value	Seqs.
Poaceae	11,554	963,585	31		Ericaceae	3,554	1,371	1
Solanaceae	2,678	280,810	14		Grossulariaceae	195	1,247	0
Fabaceae	24,505	214,599	15		Linaceae	213	848	1
Rosaceae	4,828	158,890	10		Actinidiaceae	176	788	1
Malvaceae	4,465	112,394	3		Polygonaceae	1,384	693	0
Cucurbitaceae	965	102,053	4		Aquifoliaceae	480	690	0
Arecaceae	2,522	89,828	3		Cannabaceae	102	528	2
Brassicaceae	4,060	79,650	19		Salicaceae	1,269	372	2
Euphorbiaceae	6,547	69,650	4		Canellaceae	21	344	0
Vitaceae	985	68,942	3		Sapotaceae	1,343	221	0
Rutaceae	1,730	64,431	2		Papaveraceae	920	132	0
Amaryllidaceae	2,258	63,376	0		Myrtaceae	5,970	111	2
Anacardiaceae	701	45,283	0		Urticaceae	1,465	99	0
Musaceae	78	44,859	3		Lecythidaceae	341	67	0
Asteraceae	23,600	37,734	4		Orchidaceae	27,801	9	2
Convolvulaceae	1,296	26,797	1		Lamiaceae	7,886	0	1
Amaranthaceae	2,052	25,548	4		Apocynaceae	5,556	0	1
Dioscoreaceae	653	20,858	0		Araceae	3,368	0	1
Oleaceae	688	19,467	1		Gesneriaceae	3,122	0	1
Pinaceae	255	19,268	5		Primulaceae	2,788	0	2
Rubiaceae	13,673	16,060	1		Caryophyllaceae	2,456	0	2
Juglandaceae	89	15,650	1		Plantaginaceae	1,614	0	6
Theaceae	370	12,871	0		Moraceae	1,217	0	1
Bromeliaceae	2,929	11,618	1		Thymelaeaceae	938	0	1
Asparagaceae	200	11,453	0		Rhamnaceae	839	0	1
Apiaceae	3,257	8,666	1		Meliaceae	669	0	1
Fagaceae	1,101	7,805	2		Capparaceae	449	0	1
Pedaliaceae	67	4,642	1		Lentibulariaceae	312	0	2
Caricaceae	47	4,054	1		Phrymaceae	199	0	1
Ebenaceae	751	2,811	1		Zosteraceae	23	0	1
Betulaceae	234	2,667	1		Nelumbonaceae	2	0	1
Piperaceae	2,658	2,478	0		Amborellaceae	1	0	1
Zingiberaceae	1,587	2,430	0

**Table 3:** Data set on order, including species abundance, economic value, and number of sequenced genomes

		Total value					Total	
Order	Species	(Million $)	Seqs.		Order	Species	Value	Seqs.
Poales	18,000	975,203	32		Lamiales	24,000	24,109	13
Solanales	4,080	307,607	15		Dioscoreales	1,040	20,858	0
Fabales	25,794	214,599	17		Pinales	550	19,268	5
Rosales	7,700	159,517	14		Ericales	8,000	18,128	5
Malvales	6,000	112,394	5		Gentianales	17,000	16,060	2
Sapindales	5,700	109,714	3		Apiales	5,489	8,666	1
Cucurbitales	2,600	102,053	4		Alismatales	4,500	4,408	2
Arecales	2,600	89,828	3		Piperales	4,090	2,478	0
Brassicales	4,450	89,705	21		Saxifragales	2,500	1,247	0
Asparagales	26,000	74,838	2		Aquifoliales	536	690	0
Malpighiales	16,000	70,871	7		Canellales	136	344	0
Vitales	850	68,942	3		Rannunculales	2,830	132	0
Zingiberales	2,100	47,288	3		Myrtales	11,000	111	2
Asterales	27,500	37,876	4		Proteales	1,060	0	1
Caryophyllales	11,155	26,241	6		Amborellales	1	0	1
Fagales	1,900	26,123	4					

An overall summary of the data is presented in Table 4. Of note is the order Poales with 32 genomes sequenced, 31 in the family Poaceae (grasses) plus pineapple. For 15 families with species of economic value, we found no genome sequences have as yet been published, most of them in the six orders containing species of economic value but with no published sequences.

**Table 4:** Descriptive statistics for families and orders

Families					
	Mean	Median	Std dev	Min	Max
Genomes sequenced	2.65	1	5.02	0	31
Value (USD millions)	40,288	2,429	127,325	0	963,585
No. of species	3,077	1,217	5,577	1	27,801
Orders					
	Mean	Median	Std dev	Min	Max
Genomes sequenced	5.65	3	7.35	0	32
Value (USD millions)	84,816	26,122	179,613	0	975,203
No. of species	7,908	5,450	8,531	1	27,500

## Results

### Analysis

The first question we asked in a regression analysis was: among the taxa (families and orders) containing at least one sequenced genome and/or at least one species of economic value, what is most important in determining the number of genomes sequenced, the biological salience of the taxon in terms of the total number of species it contains, i.e., abundance, or the aggregate economic value of the taxon. More precisely, for the response, or dependent, variable, we used the number of distinct species sequenced in the taxon (family or order). The two “independent”, or predictor, variables were:

- total agricultural value of the taxon, and
- species abundance in the taxon.

The aggregate value variable is a direct measure of the effect of economic inducement to sequence genomes in the taxon. The species abundance variable should reflect the importance of more scientific criteria, as a significant effect would suggest that sequencers are trying to investigate genomes that represent a larger number of same-family or same-order species, and hence feed into an evolutionarily well-distributed sample for eventual comparative goals.

An unusual aspect of this model is that we do not include families or orders that have no sequenced genome nor any species of economic value. This was largely a question of avoiding the collection of abundance data on many hundreds of families with no genomes sequenced and no economic value, and having them swamp the effect of the families of more interest. Nevertheless we will return to this question in the next section of this paper.

These results show a dominant effect of the economic importance of the species in a taxon, but also an unmistakeable effect of the species abundance of that taxon.

**Fig. 1:**
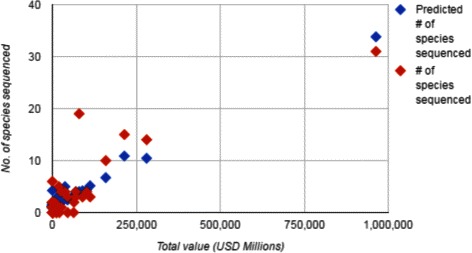
Total value (USD Millions) line fit plot, for family data

While the proportion of the variance explained is considerable (77 % for families, 70 % for orders), Fig. 1 shows that much of the variance appears, caused by a single point, representing the effect of the Poaceae, valued at over $900,000,000,000, while the next biggest value is less than $300,000,000,000. Repeating the analysis without this family gives the regression in Tables 5 and 6.

**Table 5:** Regressions of number of sequenced genomes in a taxon as a function of total value of species in that taxon and the number of species in the taxon

	Data set			
	Family		Order	
Intercept	0.9656	*p*<0.01	1.7453	*p*=0.1
Value (USD millions)	0.0000327	*p*<10^−8^	0.0000313	*p*<10^−6^
Abundance	0.000119	*p*<.05	0.000158	*p*=0.1
*R* ^2^	0.77		0.70	
No. of observations	65		31

**Table 6:** Regressions in Table 5 repeated without family Poaceae and order Poales

	Data set			
	Family		Order	
Intercept	0.6818	*p*=0.05	1.7453	*p*<0.5
Value (USD millions)	0.0000489	*p*<10^−8^	0.0000507	*p*<10^−4^
Abundance	0.00008719	*p*<.12	0.000141	*p*<0.13
*R* ^2^	0.52		0.52	
No. of observations	64		30	

**Fig. 2:**
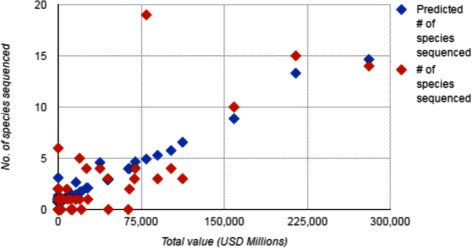
Total value (USD Millions) line fit plot, without Poaceae

We see that the economic variable, measuring total value of the order, remains highly significant, but the abundance variable recedes in significance, although the trends remain much the same. Figure 2 shows how the association of value with sequencing activity is conserved even without the Poaceae. A clear outlier is the Brassicales, with 19 genomes sequenced. This family include the genus *Arabidopsis*, containing the first flowering plant to have its genome sequenced, *Arabidopsis thaliana*, plus many closely related plants whose scientific comparison builds on the many functions and structure known first in this model plant. Brassicales also contains the intensively studied genus *Brassica*, containing many genomes of great agricultural interest - the mustards, cabbage, turnip, radishes, canola, some of which in turn have many diverse cultivars.

### Unexplored families and orders

As mentioned previously, the regression does not really do justice to the effect of species abundance, i.e., taxon size. Only those families and orders containing sequenced genomes and/or containing species of economic value, were included in the study. The large majority of families, well more than three hundred, were thus not included, as were more than half of the orders.

**Fig. 3:**
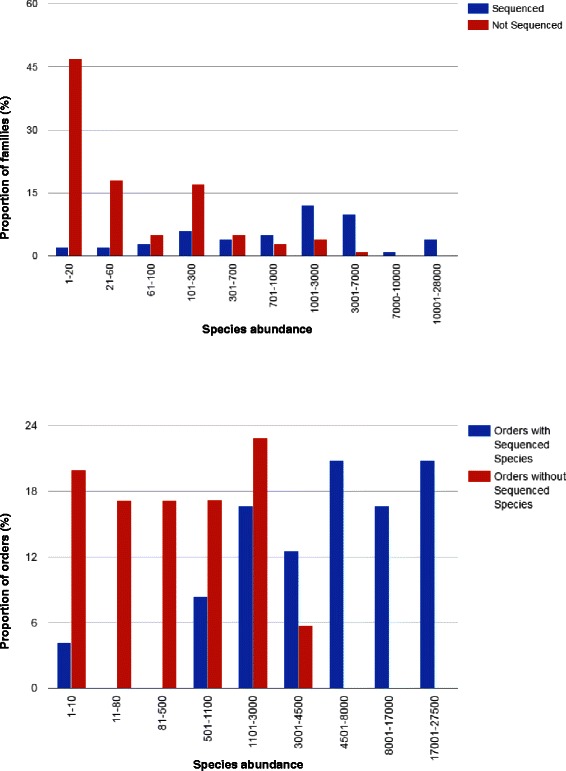
Distribution of sequenced genomes among families and orders as a function of species abundance

To compensate for this bias, we randomly sampled 100 families without genome sequences, and compared their species abundance with those in our regression study. Similarly, we calculated the species abundance for 35 angiosperm orders not in the regression study. The results appear in Fig. 3.

It is clear from the figure that the distribution of the number of sequenced genomes per taxon is not the result of a random sampling over all flowering plant species; otherwise more than half of the sequences would be in the single category of largest families. At the same time, the results do not reflect a random sampling of all the genomes; otherwise the proportion of families of a given size containing sequenced genomes would be the same as the overall proportion of families.

To summarize, the genomes that have been sequenced are concentrated in the larger families, but they are spread out to some degree among smaller families as well. This suggests that the choice of genome is motivated to some extent by the interest of the botanical community and by the specializations of PI’s and by the search for novel and diverse results.

### The next target

The strategy for choosing a genome to sequence has evolved over the years. We can ask to what extent this strategy has been directed by economic interests versus broader scientific criteria, by examining each taxon to see when its first genome was sequenced.

**Fig. 4:**
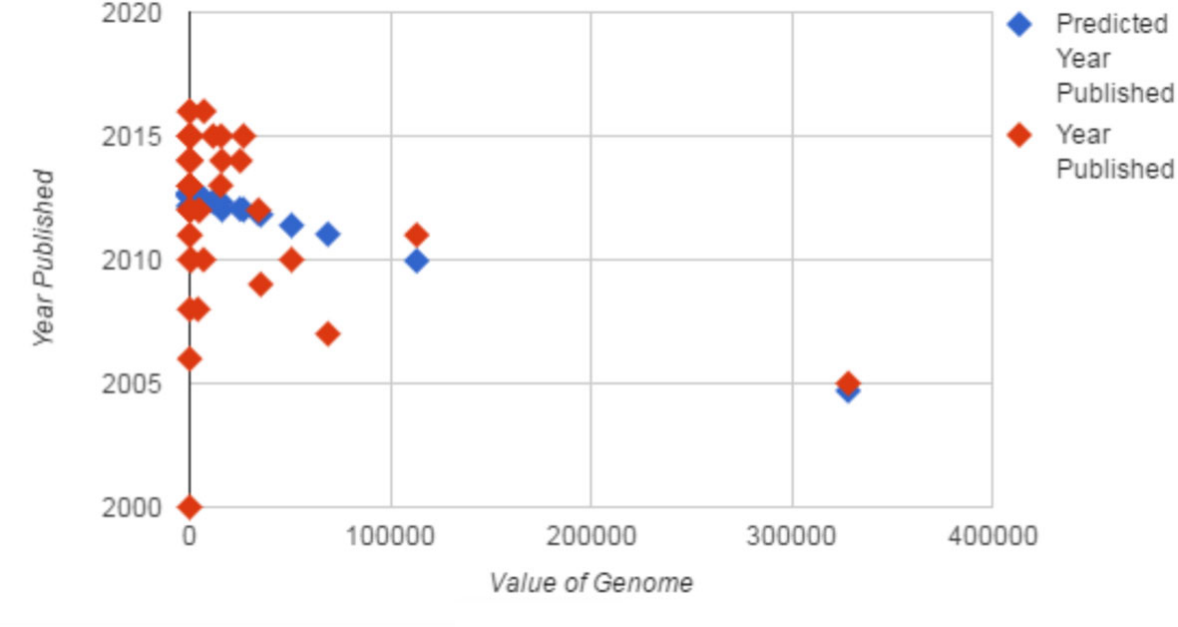
Line fit plot of year of first sequenced genome in a family versus total value (USD Millions)

Table 7 shows a regression in which the economic value of the family does have a small but significant effect on early choice of a genome to sequence. However, this regression explains little of the variance, as illustrated in Fig. 4.

**Table 7:** Regressions of year of first sequenced genome in a family as a function of total value of species in that taxon and the number of species in the taxon

Intercept	2012.7	*p*<10^−8^
Value (USD millions)	-0.0000236	*p*<0.01
Abundance	-0.0000202	*p*<0.8
*R* ^2^	0.15	
No. of observations	50	

## Discussion

### Data considerations

There are several issues regarding our data collection. The forestry data compiled was not recent or reliable [15]. These products are usually classified only by the type of consumer product (pulp, hardwood, paper …) and rarely by tree species. Furthermore, countries do not always readily provide data on forestry production, more particularly which trees are being cut down, but simply what secondary or tertiary product they are being turned into [14].

Another problem in calculating forestry data is that they are presented country-by-country as exports or imports. We were able to aggregate all the export figures to arrive at worldwide values per species, but this does not take into account internal consumption, which is certainly very important in many producing countries. This means that our estimates of total value for the forestry taxa are systematically biased downward.

After we completed our research, at the Plant and Animal Genomes Conference in January 2016 [18], we learned from posters of several plant genomes that had been recently sequenced: mango, onion, pistachio, and others. It was too late to include these in this research, as were the many others reported in the interim. Our freely accessible database (http://216.48.92.133/Softwares/PlantGenomes/index.htm), however, does list these. The integrated taxonomic/economic database is a main contribution of this work, and we intend to update and maintain it for the foreseeable future. Currently, aside from the taxonomic, economic and bibliographic information on each species, we also note the date the sequence was published. In the future, other tracks may be added, such as a categorization of the economic sphere: nutritional, medicinal, chemical, horticultural, forestry, etc.

### On the choice of genomes

We have shown that the choice of plants being sequenced is most heavily influenced by the economic value of the family and order to which it belongs. There is also a significant effect of taxon size, but sequenced genomes are not distributed randomly among the 200,000 angiosperm species, as organized taxonomically and phylogenetically by botanists. Instead, many genome sequence represent smaller orders and families.

There are many potential explanations for choice of genome beyond those we have explored. We have shown that economic value has an effect on early sequencing, but this result is weak. That many “model” plants have been sequenced is not really explanatory, in that it just displaces the question of choice from genomics to an earlier stage of collaborative research; once a species’ status as a model is accepted, it is almost surely destined to be sequenced.

Outside of model plants, the economic status of plants in particular regions is a major motivation; one only has to look at the publication dates for the two grapevine sequence papers, or the two cacao genome papers, or the two rice papers, to infer that some major competition for publication priority was at work. One aspect of this in crop plants is the urgency of breeding programs in the face of fast-moving pathogens and climate change.

On the purely scientific side, however, the focus on crops has important implications for generalizations about plant genomes, since human choices of plants to harvest (and cultivate) will have incurred many biases with respect to phenotypic characteristics and hence of genomic properties, and recent genomic evolution will also have been affected by human agricultural practices. Fortunately, biologists have been motivated to sequence the genomes of many non-crop plants.

Small genome size is a frequent inducement for a genome sequence project. Similarly the existence of a double haploid genome or other genome with highly reduced heterozygosity makes sequencing easier. On the other hand, a species with no economic value, geographically restricted, and no historical involvement with human settlements, may, despite possible difficulties with sequencing, reveal insights into the natural processes of evolution without the distortions introduced by human intervention in breeding, cultivation and environment. Finally, the scientific novelty or unusual phenotypic or ecological characteristic of a species may make it a candidate for genome sequencing. This is particularly pertinent as major journals are increasingly reluctant to publish genome sequence papers unless it reports something strikingly different and widely interesting aside from the details of the sequencing.

Many of these factors could eventually be entered in our database, leading to further understanding of the genome sequencing enterprise across the flowering plants.
